# Case report: Near-complete response to neratinib-based treatment in HR-positive *HER2*-amplified metastatic breast cancer refractory to trastuzumab deruxtecan

**DOI:** 10.3389/fonc.2024.1484750

**Published:** 2025-01-21

**Authors:** Ünal Metin Tokat, Ashkan Adibi, Esranur Aydın, Şevval Nur Bilgiç, Eylül Özgü, Onur Tutar, Mutlu Demiray

**Affiliations:** ^1^ Precision Oncology Center, Medicana Health Group, Istanbul, Türkiye; ^2^ Division of Cancer Genetics, Department of Basic Oncology, Institute of Oncology, Istanbul University, Istanbul, Türkiye; ^3^ Department of Internal Medicine, Cerrahpasa Faculty of Medicine, Istanbul University, Istanbul, Türkiye

**Keywords:** breast cancer, precision oncology, cancer genomics, HER2 antibody-drug conjugates (HER2 ADC), trastuzumab deruxtecan, neratinib, molecular tumor board (MTB)

## Abstract

Breast cancer (BC) is the leading cause of cancer-related mortality among women. The backbone of first-line treatment in HR+/HER2+ BC is dual anti-HER2 blockade combined with taxane chemotherapy. Although this regimen exhibits high rates of response and disease control in both HR+ and HR− cohorts, some patients could have intrinsic or develop acquired resistance to trastuzumab and/or pertuzumab. Here, we achieved a near-complete response in HR+ *HER2*-amplified and overexpressing metastatic BC twice through molecular tumor board (MTB) discussions: initially, with trastuzumab deruxtecan (T-DXd) when HER2 IHC was positive, and, then, with neratinib plus fulvestrant plus paclitaxel when IHC was negative. Our case presents *GATA3* and *NOTCH2* mutations, *MCL1* and *CKS1B* amplifications, as well as *ERBB3/KRAS* overexpression and ER signaling as potential new mechanisms of resistance to T-DXd. Furthermore, we demonstrated that triplet combination could induce a remarkable response in the T-DXd–refractory setting, which could be explored in future clinical trials in HR+ and HER2-activated (by RNA or protein overexpression, amplification, and mutation) patients. Our case also highlights the importance of the MTBs to dynamically and reactively manage the course of disease and treatment on a per-patient basis.

## Introduction

Breast cancer (BC) is the most frequently diagnosed cancer worldwide according to GLOBOCAN estimates (1), and the primary reason for cancer-related deaths among women (2). Hormone receptor (ER/HR)–positive patients constitute the vast majority (75%–80%) of all BC cases (3). Moreover, at least 50% of the Human Epidermal Growth Factor Receptor 2-positive (HER2+) tumors also express HRs, influencing response to anti-HER2 agents (4, 5).

The HR status is often not a primary concern for the treatment of HER2-enriched BC as the current standard of care [taxane plus trastuzumab plus pertuzumab (THP)] is clinically active in both HR+ and HR− subgroups (6, 7). Multiple HER2 Tyrosine Kinase Inhibitors (TKIs) [with anti-HER2, chemotherapy, or endocrine therapy (ET)] or two HER2 antibody-drug conjugates (ADCs), namely, trastuzumab emtansine (T-DM1) and trastuzumab deruxtecan (T-DXd), are also approved in the later lines. Although these drugs are indicated in BC based on Immunohistochemistry (IHC) and/or IHC/*in situ* hybridization (ISH) positivity, tumors may exhibit alterations at mRNA level, which may not be always concordant with IHC/ISH and may lead to differential clinical outcomes. Of the approved agents, T-DXd stands out as it showed unparalleled efficacy in previously treated HER2+ BC in the DESTINY-Breast01/02 trials (8, 9) and was superior to T-DM1 in the DESTINY-Breast03 (10). Moreover, T-DXd has recently received accelerated approval in patients with metastatic or unresectable HER2+ solid tumors who have received prior systemic treatment and have no other satisfactory options. Furthermore, DESTINY-Breast09 phase 3 trial assessing T-DXd ± pertuzumab (against THP) in the first-line treatment of HER2+ metastatic BC (MBC) is currently ongoing. However, data on progression and resistance to T-DXd remain limited. Neratinib, an irreversible pan-HER inhibitor, with chemotherapy, anti-HER2 or HER2 ADC consistently yielded favorable patient outcomes in the trastuzumab-refractory patients (11–14). Accordingly, it may also offer benefit in T-DXd–refractory setting through combinatorial approaches.

Here, we report a patient with HR+/*HER2*-amplified MBC who achieved a near-complete clinical response to trastuzumab deruxtecan but progressed after approximately 9 months. Upon progression on T-DXd, we observed rapid and near-complete resolution of the liver metastases through neratinib plus fulvestrant plus paclitaxel combination. This could offer an alternative therapeutic modality for HR+/*HER2*-amplified MBC in the post-T-DXd setting, which is currently not explored.

## Results

### Diagnosis, pathology, and treatment history

A 38-year-old female was diagnosed with invasive ductal breast carcinoma in 2015 and, consequently, underwent mastectomy. The surgical specimen was estrogen receptor-positive (ER+), progesterone receptor-negative (PR−), and HER2+ by IHC. She later received adjuvant anthracycline-based chemotherapy for 6 cycles, followed by tamoxifen. Bone metastases were detected approximately 1 year later, which were treated with palliative radiotherapy. The patient later received two anti-HER2–containing treatments: cisplatin, gemcitabine, and trastuzumab combination; and trastuzumab emtansine monotherapy (~8 months). Although partial responses were observed upon these regimens, it was followed by early progression. Capecitabine plus lapatinib was initiated, but the disease progressed with liver metastases, and the patient was admitted to our hospital for a personalized treatment approach.

The specimen from liver biopsy (liver segment II, Tru-cut) was ER+ (strong nuclear staining, 80%), PR− (no nuclear staining), and HER2− (no membranous staining, HercepTest score of 0) by pathological assessment in September 2023. Ki-67 proliferation index was 20% (at G1M phase by MIB-1 antibody). It exhibited carcinomatous infiltration consistent with metastatic breast ductal carcinoma. The tumor infiltrating the liver was characterized by low nuclear grade (modified Black I), low mitotic activity, and diffuse tubule formation (histological grade II), although it did not exhibit minimal distortion/deformation.

### CGP-guided treatment

**Figure 1:**
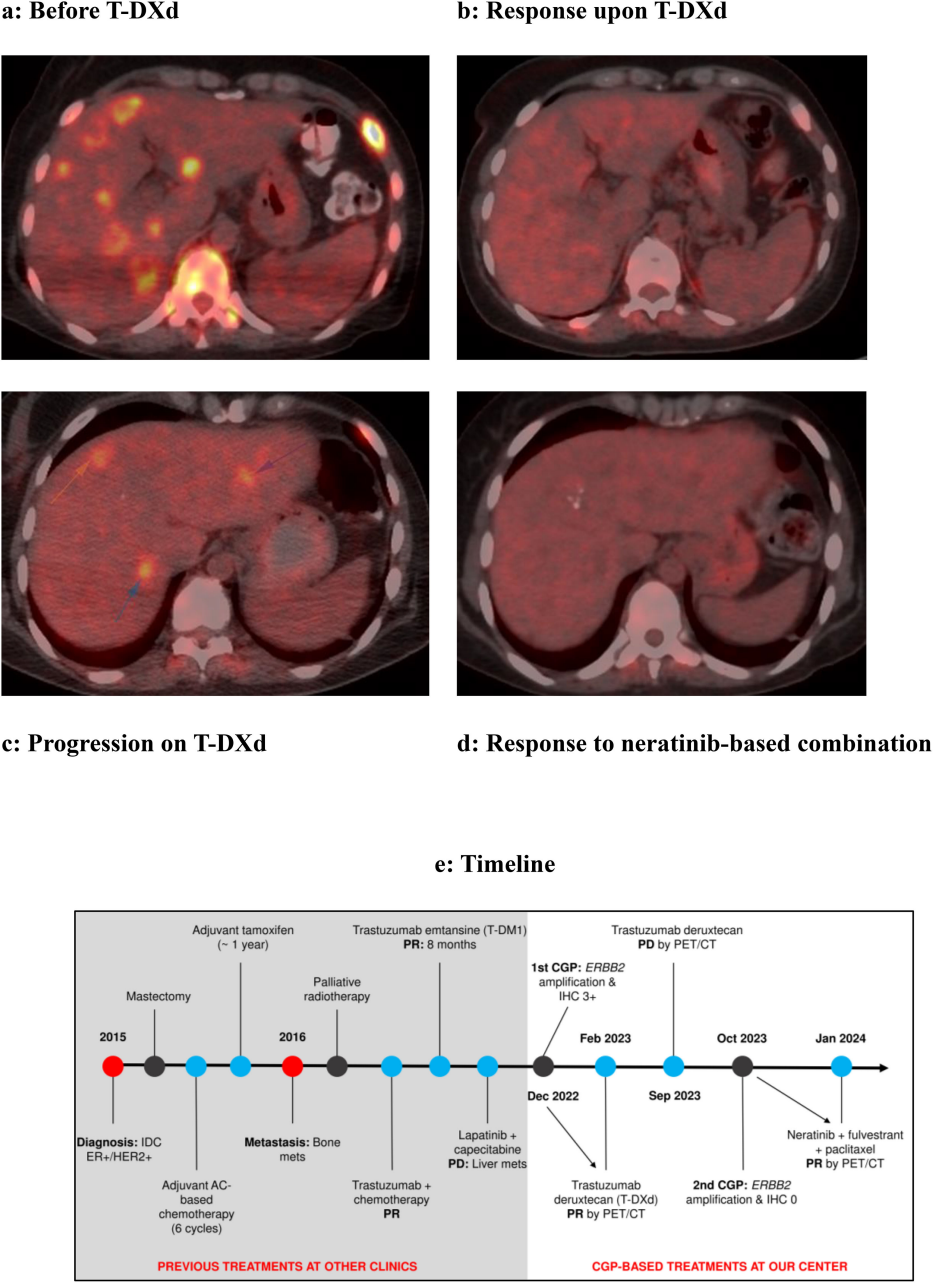
Near-complete responses in an HR+/*HER2-*amplified metastatic breast cancer patient, initially with trastuzumab deruxtecan (HER2 IHC–positive) and then with a neratinib-based combination (IHC-negative). **(A)** The December 2022 PET result showed multiple lesions with FDG uptake in the liver parenchyma consistent with metastasis. **(B)** The PET scan in February 2023 after T-DXd treatment did not reveal any lesions with significant FDG uptake. **(C)** In the September 2023 PET result, liver parenchyma shows several lesions with FDG uptake in the right and left lobes, consistent with metastasis (arrows). **(D)** In the January 2024 PET examination, no lesions showing FDG uptake could be detected in these lobes. **(E)** Disease and treatment timeline.

The initial comprehensive genomic profiling (**Table 1**) using the liver biopsy (Tempus, December 2022) detected *GATA3* P409fs (c.1223_1224insT; VAF: 11.9%) mutation as well as copy number gains (CNGs) in *ERBB2 (HER2*, 11–13 copies, IHC-positive), *ABBC3* (12), *FGF3* (11), *FGF4* (11), *MCL1* (9), *PPM1D* (≥20), *RARA* (11), *RPS6KB1* (≥20), and *TOP2A* (15). Tumor mutational burden was 3.7 muts/Mb, microsatellite instability status was stable, and Programmed death ligand 1 (PD-L1) tumor proportion score (TPS) and combined positive score (CPS) were <1% and <1, respectively. Transcriptomic profiling [research use only (RUO)] reported overexpression (OE) of *ERBB2*, *TOP2A*, *AR*, and *MAPK3* but underexpression (UE) of *CDKN2A*. Considering that the patient exhausted all the standard treatment options as well as *ERBB2* amplification with OE, we recommended trastuzumab deruxtecan (5.4 mg/kg) and achieved a near-complete response (**Figures 1A, B**).

**Table 1:** Comparison of the genomic profiles (liver metastasis) before and after trastuzumab deruxtecan treatment.

Comprehensive genomic profiling results by Tempus CDx assay		
Biomarker	1st test (December 2022)	2nd test (October 2023)
TMB (muts/Mb)	3.7	8.4
MSI	Stable	Stable
PD-L1 TPS (%) - CPS	<1 - <1	<1 - 1
HRD status (gLOH %)	ND	HRD - 15.3
Alteration (VAF% or copies)	1st test	2nd test
*ERBB2* copy number gain (CNG)	11–13	9
*GATA3* P409fs (c.1223_1224insT)	11.9	25.0
*NOTCH2* S2395*	ND	11.3
*ABCC3* CNG	12	9
*CKS1B* CNG	ND	16
*FGF3* CNG	11	ND
*FGF4* CNG	11	ND
*MCL1* CNG	9	9
*PPM1D* CNG	≥20	12
*RARA* CNG	11	8
*RPS6KB1* CNG	≥20	12
*TOP2A* CNG	15	10
RNA expression	1st test	2nd test
*ERBB2*	Overexpression (OE)	OE
*AR*	OE	OE
*CDKN2A*	Underexpression (UE)	ND
*ERBB3*	ND	OE
*KRAS*	ND	OE
*MAPK3*	OE	ND
*TOP2A*	OE	OE

HRD, Homologous recombination deficiency; ND, Not detected.

The second CGP test (Tempus, October 2023, **Table 1**) from a new liver biopsy revealed *GATA3* P409fs (c.1223_1224insT, VAF of 25.0%) and *NOTCH2* S2395* (c.7184C>G, VAF of 11.3%), and CNGs in *ERBB2* (9 copies, IHC-negative), *ABCC3* (9), *CKS1B* (16), *MCL1* (9), *PPM1D* (12), *RARA* (8), *RPS6KB1* (12), and *TOP2A* (10). The TMB was 8.4 muts/Mb, and microsatellite instability status was stable. PD-L1 TPS and CPS were <1% and 1, respectively. Homologous recombination deficiency status was negative (= not detected, LOH score of 15.3%, where threshold is 21%). Transcriptomic profiling reported *ERBB2*, *ERBB3*, *TOP2A*, *AR*, and *KRAS* OE.

Because we have previously achieved a near-complete response following T-DXd (**Figures 1A, B**) before progression within 9 months (**Figure 1C**), and it is important to target HER2 even after progression, we switched to HER2 TKI neratinib combined with fulvestrant and paclitaxel. The patient responded quickly and remarkably to this combination with a near-complete regression of the liver metastases (**Figures 1C, D**). The treatment was well-tolerated with grade 2 asthenia and decreased appetite. The initial dose for paclitaxel was 80 mg/m^2^/week, but we switched to 120 mg/m^2^ every 2 weeks to manage asthenia. The majority of the patients with neratinib-associated diarrhea experience it within the first month (15), but the patient did not report diarrhea, potentially owing to drug dose modifications and upfront loperamide use (3 days). The neratinib dose was selected as 120 mg/day (three 40-mg tablets), instead of the regular 240 mg/day (six 40-mg tablets) dose indicated in combination with capecitabine for advanced or metastatic BC (A/MBC). The 120 mg/day dose is usually utilized in the dose escalation regimen; however, we did not increase it to avoid tolerability issues. We did not modify fulvestrant dose (500 mg). The disease and treatment timeline is provided in **Figure 1E**.

## Discussion

The standard first-line treatment in HR+ and HER2+ BC consists of dual anti-HER2 in combination with taxane chemotherapy. Nevertheless, many patients still do not benefit from this regimen due to *de novo* or acquired resistance (16). In these patients, T-DXd could be primarily considered based on results from multiple phase III clinical trials. However, treatment options for patients with progression on or after T-DXd are limited and not currently explored in the clinical trials (17), except for a French multicenter retrospective study testing tucatinib, trastuzumab, and capecitabine (18). To the best of our knowledge, our case represents the first example that neratinib and fulvestrant–containing treatment could be highly efficacious in the T-DXd refractory HR+ MBC (**Figure 1**).

Tumors may have alterations at DNA, RNA, and protein levels, which may not always be consistent and may result in differential clinical outcomes. A recent study in multiple cancer types reported that *ERBB2* mRNA OE (33.3%) was much more common than cases with IHC-positive (9.3%) and amplification (4.1%) (19). In patients with all three tests, only 7.5% were positive, whereas more than 60% were negative for all the tests, where 20% of mRNA OE cases were negative for amplification and IHC. In line, whereas *HER2* mRNA OE was retained in two different CGP tests in our case, HER2 IHC changed from 3+ to 0 upon 9 months of T-DXd treatment. Another study comparing IHC and RNA reported a strong positive correlation, but baseline RNA levels were significantly higher in responders to T-DXd compared to the non-responders (20). More importantly, *ERBB2* mRNA OE exhibited a significant correlation with HER2 protein levels in the ER-negative tumors but not in the ER-positive tumors (21), in keeping with our patient (ER+) having *ERBB2* OE in the second CGP test but negative HER2 IHC. Moreover, mRNA level could define HER2 dependency and response to treatment in certain tumors, reported for a cholangiocarcinoma patient (19) and a gastric cancer patient with high *ERBB2* mRNA (22). In addition, although *HER2* mRNA level, IHC and adjusted plasma copy number were all significant predictors of response to T-DXd in the phase 2 DESTINY-Gastric01 (DG01) trial, mRNA outperformed the other parameters in terms of separating patients by overall response rate (ORR) and median overall survival (OS) (23). Still, HER2 protein level could explain differences between response to HER2-based approaches in many other cases, especially among IHC 3+ versus IHC 0 and, to some extent, those with equivocal IHC (2+). This is more prominent in the case of anti-HER2 agents as compared to HER2 ADCs or TKIs. For example, patients with BC with *HER2* amplification but equivocal expression experienced a worse prognosis with a lower response rate following anti-HER2 treatment. This was independent of the *HER2* copy number. These specimens were also enriched for trastuzumab resistance and ER signaling genes. DESTINY-CRC01 (DCRC01) trial of T-DXd in CRC patients reported that higher levels of HER2 biomarkers in baseline tissue and liquid biopsies, including HER2 status (IHC/ISH), HER2/CEP17 ratio, HER2 ISH signals, HER2 H-score, plasma *HER2* [Human Epidermal Growth Factor Receptor 2 (ERBB2)] amplification status, *HER2*-adjusted plasma copy number, and HER2 extracellular domain correlate with antitumor activity (24). The ORR for patients with IHC 3+ was 57.5%, whereas it was only 7.7% for IHC 2+/ISH+. The median progression-free survival (PFS) and overall survival (OS) were also dramatically more favorable in the former versus the latter group, potentially explaining near-complete response to T-DXd when IHC was 3+ in our patient, and it was 0 when disease progressed on T-DXd. In preclinical models, HER2 homodimer level was associated with response to trastuzumab compared to EGFR: HER2 and HER2:HER3 heterodimers (25). *ERBB2* and *ERBB3* OE in our post-T-DXd specimen (**Table 1**) could cause increased HER2:HER3 heterodimers, thus potentially limiting therapeutic efficacy. DESTINY-Breast04 (DB04) and DESTINY-Breast06 phase 3 trials reported that T-DXd provided significantly longer PFS even in previously-treated HR+ HER2-low (IHC 1+ or IHC 2+/ISH−) and HER2-ultralow (IHC 0 with membrane staining; IHC > 0 <1+) A/MBC (26, 27). Importantly, however, HER2 expression significantly decreased at the time of resistance to T-DXd, although there was no clear difference in T-DXd uptake in the phase 2 DAISY trial (28). In our case, *ERBB2* OE was present in pre- and posttreatment specimens, but decrease of copy number from 11–13 to 9 in the posttreatment could be indicative of reduced expression (**Table 1**). The trial also reported that best overall response (BOR) confirmed was 70.6% and 29.7%, median PFS of 11.1 months and 4.2 months, and median of OS 31.2 months and 12.1 months in Cohort 1 (IHC 3+ or ISH+) and Cohort 3 (IHC 0), respectively (29). This is consistent with our case, considering near-complete response when IHC 3+ and progression when IHC 0. The duration of response in the Cohort 1 of the DAISY trial was also similar to our case (9.7 vs. 9 months).

Genomic profiling of patients with *ERBB2*-positive BC before and after trastuzumab plus chemotherapy revealed that *NOTCH2* and *GATA3* mutations, along with others, were clonally enriched and/or acquired in the post-treatment samples (30), which could be also relevant in our case. The *GATA3* P409fs variant allele frequency increased from 11.9% to 25.0% in the second CGP test, whereas the *NOTCH2* mutation was only detected in the post-trastuzumab deruxtecan biopsy (**Table 1**). *GATA3* mutations were identified in approximately 15% of HR+ patients (31, 32). P409fs was the most frequent variant in the SCAN-B BC cohort, and more common in the luminal/HER2− or ER+ subtype (33, 34). Although the initial tumor specimen from our patient was reported to be ER+/HER2+, the refractory tumor was ER+/HER2-. Frameshift *GATA3* mutations could lead to either truncation or elongation of the protein, with potentially distinct downstream consequences. Accordingly, GATA3 could function as tumor suppressor or oncogene depending on the mutation (31, 32). Therefore, its role should be assessed on a per-patient basis. P409fs mutation results in an elongated protein product, and such extension mutations were associated with shorter disease-free survival (DFS) compared to other *GATA3* mutations among The Cancer Genome Atlas (TCGA) Program BC patients (35). *GATA3* mutations are associated with an increased *GATA3* mRNA level (32). A positive correlation was also reported between GATA3 mRNA and protein levels in BC (36). Importantly, GATA3 IHC is routinely employed to ascertain breast or urothelial origin in carcinomas of unknown primary (37). GATA3 positivity was significantly associated with ER+ and p53 wild-type pattern in BC. *GATA3* mutations and/or expression were shown to cause resistance to certain endocrine therapies while retaining sensitivity to others. For instance, patients with *GATA3-*mutant ER+ MBC had worse PFS and OS compared to those with *GATA3* WT upon treatment with selective estrogen receptor degraders alone or in combination with CDK4/6 inhibitors (38). On the contrary, these mutations were associated with sensitivity to aromatase inhibitors (36, 39–41) and tamoxifen (42–44) in diverse settings. Considering significant outcomes in patients with HR+/HER2-low MBC upon T-DXd combined with anastrozole or fulvestrant in the DESTINY-Breast08 trial, T-DXd plus anastrozole could be prioritized for the HR+/HER2+ or HR+/HER2-low patients with *GATA3* mutations. Another strategy could be the use of inhibitors against other proteins to limit GATA3-driven mechanisms, as there are no approved GATA3 inhibitors despite some preclinical molecules, such as pyrrothiogatain (45). For example, GATA3 and MDM2 were found to be synthetic lethal in ER+ BC (46), and MDM2 inhibitor milademetan was tested in a phase II clinical trial (DEMETER – NCT05932667) in combination with fulvestrant in patients with *GATA3*-mutant, ER+/HER2− A/MBC but it was terminated due to financial issues. Similarly, Histone deacetylase (HDAC) inhibition was shown to impair GATA3-dependent gene transcription (47), and HDAC inhibitor (HDACi) vorinostat in combination with tamoxifen was found to be clinically active in patients with endocrine-resistant BC (48). This approach could be re-tested in trials by combining HDACi with new generation of endocrine therapies. Direct and/or indirect strategies to *NOTCH2* alterations are even less explored than *GATA3* alterations. Gamma secretase inhibitors (GSIs) or pan-NOTCH inhibitors could be utilized to target NOTCH signaling; however, GSIs predominantly function through the inhibition of NOTCH1 signaling. Moreover, whereas NOTCH1 was characterized as an oncogene in BC, the role of NOTCH2 remains elusive (49, 50). Although GSI nirogacestat was approved by FDA for desmoid tumors and could be used off-label in other cancer types including the breast, GSI activity against *NOTCH* rearrangement and mutations could display significant differences by rearrangement product and location of the mutation, limiting their use as a general strategy for all *NOTCH2* alterations. Very recently, a pan-NOTCH inhibitor RO4929097 was shown to reverse paclitaxel resistance in preclinical cancer models (51). Considering that dual anti-HER2 plus taxane and carboplatin plus paclitaxel with anti-HER2 regimens utilized in BC, such a strategy could be important in treatment-refractory patients.

Among the amplified genes, only *MCL1* did not have a decreased copy number, and *CKS1B* CNG was only detected in the post-T-DXd specimen (**Table 1**). These genes are located on the same amplicon (1q21). *CKS1B* is involved in cell cycle regulation through its interaction with cyclin-dependent kinases (CDKs). Its overexpression has been associated with decreased OS and DFS, in part due to its association with loss of differentiation, young age, and negative Estrogen receptor/Progesterone receptor (ER/PR) status (52), whereas our patient was ER+/PR−. This OE could drive apoptosis in preclinical BC models upon Polo Like Kinase 1 (PLK1) inhibition (53), which could be activated by ERBB receptors (54). Consequently, response or resistance to HER2 ADC or HER2 TKI could be improved or overcome by PLK inhibition (55). Considering the lack of approved PLK1 inhibitors and its activity in kinetochore-microtubule dynamics and mitosis, microtubule-targeting agents or mitotic inhibitors could represent alternative therapeutic options. Taxanes are regularly used with anti-HER2 agents in HER2-positive BC. Furthermore, a mitotic inhibitor-bound HER2 ADC, T-DM1 is indicated for HER2-positive unresectable A/MBC who have previously received trastuzumab and taxane. MCL1, anti-apoptotic protein in Bcl-2 family, was identified as a clinical target in both *HER2*-amplified and triple-negative BCs (56) and implicated in resistance to HER2-targeted therapies (57). In these models, however, MCL1 inhibitor (S63845) demonstrated a synergistic activity with lapatinib, trastuzumab, and docetaxel. In line, constitutive BAK/MCL1 complexes were associated with paclitaxel and S63845 response in ovarian cancer PDX models.

Neratinib is a potentially overlooked treatment option for A/MBC, especially after progression on the standard-of-care (SOC) and could be utilized in combination with other therapeutics (11–14, 58, 59). Neratinib is even under investigation with T-DXd in advanced refractory gastric cancer patients (60). The initial phase I/II trials investigating neratinib and trastuzumab combination documented their tolerability and encouraging clinical activity, with durable responses up to 10-year observed in some patients (14). In phase III NALA trial, for example, neratinib plus capecitabine resulted in significantly prolonged PFS compared to lapatinib plus capecitabine in HER2+ MBC previously treated with at least two HER2-directed regimens (59), of whom almost all or more than half received trastuzumab or T-DM1, respectively. Furthermore, phase II NSABP FB-10 trial yielded an ORR of 63% upon neratinib plus T-DM1 in the HER2+ MBC (13, 61). *ERBB2* amplification in study entry biopsies, *ERBB2* RNA levels, and loss of detectable ctDNA and *ERBB2* amplification in the ctDNA were identified as biomarkers of response. Our case is consistent with the first two biomarkers, although we do not have any data on ctDNA. Similar to T-DXd and unlike anti-HER2 agents, neratinib monotherapy could be also effective in HER2-low cases (62), explaining near-complete response in our patient despite HER2 IHC 0 with *ERBB2* mRNA OE. Moreover, whereas p95HER2 was associated with trastuzumab resistance (63), two complete responders in the NSABP FB-10 trial had high total HER2 and p95HER2 levels in the baseline tissue. The p95HER2 is also likely to limit T-DXd activity (64), which could be one of the reasons driving resistance in our patient. Resistance to HER2-targeted therapies in HR+/HER2+ tumors following an initial response could be also caused by ER overexpression and/or activation (65, 66), potentially due to extensive bidirectional crosstalk between ER and HER2 signaling. Very recently, DESTINY-Breast08 reported remarkable outcomes in chemotherapy-naïve patients with HER2-low, HR+ MBC upon T-DXd combine with anastrozole or fulvestrant. Similarly, neratinib resulted in longer DFS only in HR+/HER2+ patients, almost all of whom also received ET, in the phase 3 ExteNET trial (12). Finally, the SUMMIT phase II trial in the patients with HR+/HER2−, *HER2*-mutant MBC not only supported a synergistic activity of neratinib with fulvestrant plus trastuzumab but also proved requirement for neratinib as there were no responses with fulvestrant or fulvestrant plus trastuzumab (67). *HER2* mRNA expression did not significantly differ between partial response (PR), stable disease (SD), or progressive disease (PD) subgroups, although the sample numbers are quite low. Neratinib-containing regimen was even active in *ERBB3* co-alterations (overexpressed in our case), implicating that HER2:HER3 heterodimer activity could be blocked in these tumors. Briefly, HER2 ADCs (with different platforms or payloads) or TKIs with ET combination could be tested in future clinical trials in the HR+ patients upon progression on T-DXd (17). These trials could be designed to not only assess the mutational profile but also other omics data such as transcriptome profiling by RNA sequencing, relevant serum/plasma biomarkers and cancer-specific clinical parameters. For example, the DG01 trial presented subgroup analyses regarding HER2 IHC, *HER2* mRNA expression, plasma *HER2* copy number/amplification and gain-of-function (GOF) mutations, and serum HER2 extracellular domain (ECD) (23). The DCRC01 also included HER2/CEP17 ratio, HER2 ISH signals, and HER2 H-score (24). The future trials of the T-DXd could be designed to analyze the efficacy by multiple HER2-related parameters utilized in this trial to define their relative importance as well as the outcomes for discordant cases (such as *HER2* amplification and OE with IHC-negative status in our case). Unlike detailed analysis on the HER2, the DG01 performed subgroup analyses for only *MET*, *EGFR*, *FGFR2*, *PIK3CA*, and *KRAS*/*NRAS* based on their mutational status. Other DESTINY trials, such as Breast04 (*ESR1*, *PIK3CA*, and CDK4/6i resistance genes) (68), CRC01 (*RAS*, *PIK3CA*, and *HER2*) *(* 24), and Lung01 (*EGFR*, *KRAS*/*NRAS*, and *BRAF*) *(* 69), similarly focused on the mutational status of few genes. Accordingly, the alterations in genes with potential association to the T-DXd resistance in our case (*GATA3*, *NOTCH2*, *CKS1B*, and *MCL1*) could be highly relevant for multiple cancer types, primarily for breast, bladder, and gynecologic cancers as well as lung adenocarcinoma, lung squamous cell carcinoma, cholangiocarcinoma, and hepatocellular carcinoma [cBioPortal (70)]. However, these analyses should ideally be expanded to incorporate individual mRNA–, gene expression signature–, and even pathway activity scoring–based analyses while still considering alteration status, although the genes selected for mutational subgroup analysis in the DESTINY trials make sense given their frequency and clinical relevance on the studied cancer type. According to the results from potential studies outlined above, T-DXd combinations with HER2 TKIs (such as neratinib) and other drugs could be tested as the next step. It is important to note that these combinations should be assessed on the basis of precision oncology principles to match the right treatment with the right patient, maximize the clinical activity, and minimize the drug and financial toxicity through dose modifications and regimen scheduling.

Overall, to our knowledge, this is the first report showing the efficacy of HER2 TKI–based combination therapy in T-DXd–refractory MBC (**Figure 1**). Although T-DXd could be utilized even in HER2-ultralow patients, HER2 expression loss could impair its activity and cause disease progression. In this setting, we achieved a near-complete response with neratinib in combination with fulvestrant and paclitaxel in patients with heavily pretreated HR+, *HER2*-amplified but HER2-negative (by IHC) MBC.

## Methods

### Comprehensive genomic profiling

CGP was performed by Tempus Labs Inc. (Chicago, IL, USA) through their proprietary technology. However, the methodology could be broadly outlined as follows: Formalin-fixed, paraffin-embedded tumor tissue blocks were retrieved, and hematoxylin and eosin–stained slides were reviewed by a board-certified pathologist to confirm tumor presence and assess tumor cellularity. Only samples with ≥20% tumor cellularity were included. Blood samples were also collected as a control for germline analysis. DNA and RNA were extracted from the tissue samples using the Tempus xT proprietary extraction protocols. The concentration and quality of the extracted nucleic acids were assessed. For DNA sequencing, libraries were prepared using the Tempus xT library preparation kit. For RNA sequencing, cDNA synthesis was performed, followed by library preparation using the Tempus xT RNA-seq kit. Paired-end sequencing was performed using an Illumina NextSeq or NovaSeq platform. DNA libraries were sequenced to an average depth of 500× coverage, whereas RNA libraries were sequenced to an average depth of 100 million reads per sample. Raw sequencing data were processed using the Tempus xT bioinformatics pipeline. A detailed report was generated for each patient, summarizing the clinically relevant findings. The report included information on actionable mutations, potential therapeutic targets, and relevant clinical trials.

### Molecular tumor board

Our team in the molecular tumor board discussions consists of medical oncologists, molecular biologists, geneticists, radiologists, and, sometimes, surgeons, a pathologist, and a medical geneticist. Considering that the patient has been previously benefited from anti-HER2–based treatment, tumor specimen was still HER2-positive and remarkable responses to trastuzumab deruxtecan in multiple settings in breast and pan-cancer cohorts, we initially started treatment with T-DXd. A near-complete response was observed in the liver metastases. Upon progression, we switched to neratinib, fulvestrant, and paclitaxel combination. This was based on the following rationale: ER signaling could impair clinical activity of anti-HER2 agents, HER2 blockade is still important even in patients progressing on or after anti-HER2 treatment, neratinib could irreversibly inhibit both membranous and intracellular (if any) HER2 proteins, and mitotic inhibitor paclitaxel could help targeting *CKS1B* and *MCL1* CNGs within the combination regimen.

## Data Availability

The original contributions presented in the study are included in the article/supplementary material. Further inquiries can be directed to the corresponding authors.
